# Osteoporosis in children with chronic illness: lessons from natural history studies that guide clinical practice

**DOI:** 10.1186/1687-9856-2015-S1-O10

**Published:** 2015-04-28

**Authors:** Leanne M Ward

**Affiliations:** 1 University of Ottawa, Ontario, Canada

## 

Children with serious chronic illnesses have the potential for significant bone morbidity, particularly in the context of disorders with impaired muscle function and the need for glucocorticoid (GC) treatment. Recently, studies have shown that vertebral fractures are often the first sign of osteoporosis in this context. Longitudinal studies have taught us that vertebral fractures are most frequent in the first year of GC therapy among children with GC-treated diseases, that vertebral fractures are often asymptomatic and thereby go undetected in the absence of routine monitoring, and that even asymptomatic vertebral fractures predict an increased risk of future fractures. Other discrete clinical predictors of incident vertebral fractures are evident early in the course of GC therapy, including decreases in spine BMD Z-scores and increases in body mass index Z-scores in the first 6 months of GC therapy. Taken together, these observations highlight that the first year of GC therapy is a critical period for monitoring in order to identify bone morbidity in a timely fashion. Children with risk factors for bone morbidity other than GC therapy (such as neuromuscular disorders and immobilization) are also at increased risk for vertebral and non-vertebral fractures fractures. As such, routine surveillance for vertebral fractures (for example, with a lateral spine radiograph) is now considered a key facet of the bone health evaluation in children with chronic immobilization or GC-treated diseases.

While some risk factors for osteoporosis will be permanent (such as immobilization arising from disorders such as cerebral palsy), other children will experience transient threats to bone health. This is an important distinction, since children with transient threats have the potential for vertebral body reshaping, either spontaneously or with bone-targeted treatment such as bisphosphonate therapy. Recovery from fracture-induced deformity is growth-dependent, underscoring the importance of timely diagnosis and intervention during childhood in those with limited potential for spontaneous vertebral body reshaping.

Prevention begins with optimization of conservative measures, including physical activity, nutrition, treatment of co-morbid endocrinopathies and aggressive treatment of the underlying disease using the lowest effective GC dose. These measures may be insufficient to prevent first-time fractures in some, raising the need for bone-specific therapy in line with a secondary prevention approach. Secondary prevention is predicated upon identification of early signs of osteoporosis and intervention to prevent disease progression. Since bone-targeted treatment is typically reserved for children with overt fragility, careful monitoring to avoid advanced osteoporosis presentations is paramount. Bisphosphonates are the most commonly prescribed agents in children; however, interest in the use of novel osteo-anabolic therapy is mounting given bone histomorphometric observations that osteoporosis in children with serious chronic illnesses is typically characterized by significant reductions in bone turnover.

